# Are rare diseases overlooked by medical education? Awareness of rare diseases among physicians in Poland: an explanatory study

**DOI:** 10.1186/s13023-021-02023-9

**Published:** 2021-09-28

**Authors:** Dariusz Walkowiak, Jan Domaradzki

**Affiliations:** 1 Department of Organization and Management in Health Care, Poznan University of Medical Sciences, Przybyszewskiego 39, 60-806 Poznan, Poland; 2 Department of Social Sciences and Humanities, Poznan University of Medical Sciences, Rokietnicka 7, St., 60-806 Poznan, Poland

**Keywords:** Rare diseases, Physicians, Medical education, University curricula

## Abstract

**Background:**

During their studies, future physicians are often taught that while evaluating a patient they should first consider a common diagnosis and not a rare one. Consequently, although most physicians will face the diagnosis or treatment of a rare disease (RD) at some point in their professional lives, many assume that they might never meet a patient with a specific RD. Moreover, many physicians lack knowledge about RDs and are not prepared for caring for RD patients. Thus, the aim of this paper was to assess the awareness of RDs among Polish physicians.

**Methods:**

The study was conducted among 165 medical doctors taking their specialization courses at the Poznan University of Medical Sciences, Poland. The questionnaire assessed physicians’ knowledge about the number, examples, etiology and estimated frequency of RDs. It also checked the self-assessment of physicians competence in RDs, as well as their opinions about university curricula in this respect.

**Results:**

The study shows that while most physicians lacked basic knowledge about the etiology, epidemiology and prevalence of RDs, many had also problems with separating RDs from more common disorders. Moreover, 94.6% of physicians perceived their knowledge on RDs as insufficient or very poor and less than 5% feel prepared for caring for patients with RDs. Simultaneously, while over 83% of physicians believed that RDs constitute a serious public health issue, 17% were of the opinion that mandatory courses on RDs are not necessary in medical curricula and 6.7% were not interested in broadening their knowledge of such diseases. Most respondents derived their knowledge on RDs from university courses, scientific literature and research, as well as from the Internet.

**Conclusion:**

Since the study shows that there is a urgent need to fill the gap in physicians’ knowledge on RDs, it seems advisable that extra courses on these diseases should be added to medical curricula and physicians’ postgraduate training. Furthermore, as the Internet is the main source of information on RDs, e-learning programs and courses for all medical professionals should be organized.

## Background

Similarly to other European countries, rare diseases (RDs) are defined in Poland as the ones that affect no more than one person in 2000 [1–3]. However, although such diseases seem to be very rare, there can be observed a dual paradox of RDs. Firstly, they include conditions that are often perceived as quite ‘common’ and are not recognized as RDs, i.e. phenylketonuria, cystic fibrosis or achondroplasia. Indeed, it is estimated that from all 6000–8000 RDs, 100 conditions account for 80% of all RD patients [4]. Secondly, while taken individually RDs are rare or ultra-rare, when combined they affect a significant number of people, as approximately 6–8% of the world’s population suffer from them. This means that between 300 and 350 million people worldwide are affected, including 27–36 million in the European Union (EU) [5] and 2.3–3 million in Poland [3, 6–8]. Thus, because RDs affect a very large number of people they constitute an important medical and social challenge and an urgent public health issue [4, 9].

For this reason, in 2009 the Council of the European Union recommended that RDs should be prioritized and that all member states should adopt a national strategy for RDs [10]. Nevertheless, even though the right to healthcare of all Polish citizens is protected constitutionally and everyone is entitled to equal access to public healthcare, RDs are often overlooked or even neglected by drug makers, doctors, developers, government officials and policy makers, public health programs and news media [11, 12]. For example, although most European countries have either implemented or created national plans for RDs, Poland still has not adopted such a strategy [13, 14]. Furthermore, research conducted among (future) healthcare professionals show that neither do they possess knowledge about RDs nor feel prepared for caring for such patients [15–19]. For example, a recent study shows that while 95% of future nurses, physiotherapists and physicians assessed their knowledge about RDs as insufficient or very poor, a similar percentage did not feel prepared for caring for RD patients. Moreover, 55% of medical students did not see the need to include the RDs topics into the medical curricula [19].

This is important because during their studies future physicians often hear the following teaching pearl: ‘When you hear hoofbeats, think of horses not zebras!’ and are taught that while evaluating a patient, a physician should first consider a common and not a rare diagnosis [20]. However, the ‘Don’t think zebras’ approach reproduces the common belief in the rarity of RDs. Consequently, although most physicians will face the diagnosis or treatment of a rare disease at some point in their professional lives, many assume that they might never meet a patient with a specific RD. Meanwhile, while some RD patients are encountered on a daily basis, the peculiar character of the clinical setting (routine, lots of patients, lack of time) makes it difficult to separate rare from common diseases [21–23]. Additionally, because the vast majority of RDs are of a genetic character and affect children, many physicians believe that it is mainly geneticists and paediatricians who should be trained with regard to RDs. Meanwhile, as RDs are very complex, require multiple services and care over a long period of time, adequate care coordination is of special importance for people affected by them. For that reason the recognition of RD is important for all medical specialties. This in turn, requires the organization of a multi-professional care that should include not only primary-care physicians but also such specialists as geneticists, neurologists, psychiatrists, immunologists, paediatricians, dieticians, pharmacists or physiotherapists [23–27]. And the first step to achieve this is to assess the physicians’ educational needs and fill the gap in their knowledge on RDs. The reason for this is twofold. First, rare conditions considered jointly do affect a very large number of people in the country. Second, only a small percentage of Polish physicians, both primary and specialized care physicians, receive training in RDs during their undergraduate or postgraduate years. Consequently, there is an urgent need to raise the awareness on RDs among physicians. It is even more important as according to the Polish Ministry of Health’s declarations, one of the key points of future strategy for RDs should be the improvement of undergraduate and postgraduate education on these diseases [7].

In consequence of the physicians’ lack of knowledge and specific training, many RDs are un/misdiagnosed and RD patients struggle with an endless diagnostic and therapeutic odyssey [28]. This in turn impedes the inclusion of RD patients into society and creates many barriers that prevent the fulfilment of their basic needs, including education [29, 30]. For example, some children suffering from an RD in Australia received a proper diagnosis only after 10 or even 18 years and reported visiting more than ten physicians [31]. Similarly, the mean time of diagnosis for Polish patients suffering from Huntington disease was 10.5 years, although in some cases it was even 30 years [32]. A recent study in Ireland found that 69% of RD patients were diagnosed in adulthood [25]. Consequently, both RD patients and their families have to become self-experts in their disease and educate their physician about their (child’s) rare disease [32–35]. Thus, while it is impossible to know all the RDs that have been identified, physicians should nevertheless be prepared and trained to identify ‘the zebras’ among the horses.

General practitioners in Poland are trained at the university level during a 6-year course. While during the first 2 years of training future physicians study general medical matters, such as physiology, biochemistry and biophysics, in the following years, knowledge is directed more towards the specialization chosen. Additionally, every year students undergo compulsory internships in hospitals or other medical institutions. To get the full right to practice, physicians have to pass the National Medical Exam and complete a 13-month professional internship. After that, young physicians may choose specialist education which consists of five basic modules that correspond to the basic range of theoretical knowledge and practical skills in general medicine (general surgery, otorhinolaryngology, pathology, pediatrics and internal medicine) and a specialist module, which covers seventy seven different specialties, including clinical genetics, diagnostic laboratory, neurology, psychiatry or pediatrics, apart, of course, from those that are most common in demand.

At the same time, although future physicians in Poland are trained in molecular biology, diagnostic laboratory and clinical genetics (sometimes it also includes classes in genetic counselling) and are taught about some genetic diseases (i.e. cystic fibrosis, phenylketonuria, Huntington disease, Pompe disease, Niemann–Pick disease or sickle cell anemia), the methods and types of materials used in genetic laboratory diagnostics and the psychosocial consequences related to genetic diagnosis, there are no special courses dedicated to RDs; neither are they trained in clinical genetics ambulatories. Moreover, also postgraduate specialization courses give little or no information on RD. For example, while during some courses physicians receive one hour lecture dedicated to the National Rare Disease Plan or are taught about funding of RD treatment, most specialized courses give no information on caring for RD patients and related subjects.

It should also be emphasized that while medical education systems in various European countries are quite similar, they do differ in the way medical knowledge and skills are taught. Thus, although medical students in Poland receive very complex theoretical background, in the United Kingdom or the Scandinavian countries medical education is much more practical, medical students are often incorporated into the healthcare team and are better qualified in social skills [36].

Thus, the aim of this study was to assess the awareness of rare diseases among Polish physicians. Poland was chosen for this study for two reasons. First, although Poland is the biggest country in the Central and Eastern European (CEE) areas, it still lags behind the rest of the EU with regard to medical education on RDs. Second, as Poland is yet to implement its national plan in the field of RDs it can serve as an interesting research field for that purpose.

## Methods

The study was conducted between February 2020 and March 2021 among medical doctors taking their specialization courses at the Poznan University of Medical Sciences, Poland. The process of elaborating the questionnaire followed the guidelines of the European Statistical System [37]. First, during an online focus-group meeting four general practitioners and one sociologist elaborated a list of important issues on RDs. Second, a preliminary questionnaire was assessed by two external reviewers: one physician and one sociologist. Third, the questionnaire was pre-tested via an online platform with four other physicians. Based on the pilot study, three question were reformulated. The physicians. Based on the pilot study, three question were reformulated. The final version of the questionnaire was again evaluated by two other external reviewers of the same specialties. Additionally, ethics approval and research governance approval were obtained from the PUMS Bioethics Committee (KB—1018/18). After receiving the final approval, an online survey was distributed to medical doctors taking their specialization courses at PUMS.

The questionnaire itself was divided into three sections. The first group of questions referred to the definition, aetiology and estimated prevalence of RDs worldwide, in the EU and in Poland. In this part of the questionnaire physicians were also asked to separate RDs from more common disorders from a list comprising twenty eight diseases. The second section addressed physicians’ education about RDs and their self-assessment of their knowledge and competence in the field of these diseases. The last section referred to physicians’ demographic data.

The data collected in the questionnaires were verified and checked for completeness, quality and consistency. Then they were coded and exported into the statistical packages JASP (Version 0.14.1.0) and Dag-stat [38]. The results were presented as descriptive statistics. A Likelihood Ratio Chi-square was used to assess differences in the distribution of answers among the groups. The odds ratio (OR) was calculated to compare groups. The 95% confidence interval (95% CI) was calculated to estimate the precision of the OR. A 5% level of significance was used for all hypothesis tests.

## Results

Out of all the 235 physicians approached, 165 (70.2%) completed the questionnaire (Table 1). Physicians who refused to participate in the study did so because they lacked an interest in the study and/or were unwilling to discuss their knowledge on RDs. Our study group comprised 107 females (68.8%) and 58 males (31.2%), all of Polish origin. The vast majority of respondents were either in their thirties (n = 97, 57%) or younger (n = 47, 28.5%). 55.2% of physicians declared working in the profession for up to 5 years, while 23% had from 6 to 10 years of professional experience and 10.9% had practiced form more than 15 years. While 75.2% of respondents declared having met a person suffering from an RD during their professional career, 22.4% have not. Additionally, 11.5% of physicians reported having a person suffering from an RD in their family.

**Table 1:** Socio-demographic characteristics of physicians

Characteristics	N (%)
Sex	
Female	107 (68.8)
Male	58 (31.2)
Age	
Less than 30	47 (28.5)
30–39	94 (57)
40–49	15 (9.1)
50–59	8 (4.8)
60–69	1 (0.6)
Years of professional experience	
Less than 1	6 (3.6)
1–5	91 (55.2)
6–10	38 (23)
11–15	12 (7.3)
16–20	5 (3)
More than 20	13 (7.9)
Have you ever met a person suffering from an RD	
Yes	124 (75.2)
No	37 (22.4)
I do not know	4 (2.4)
Is anyone in your family suffering from an RD?	
Yes	19 (11.5)
No	142 (86.1)
I do not know	4 (2.4)

All but one respondent declared having heard the term ‘rare disease’ (Table 2). However, only 18.2% of physicians knew the frequency of the prevalence of RDs and 20.6% correctly estimated the number of RDs. Slightly more than one-eighth of the physicians assessed properly the number of people suffering from an RD in Poland (15.8%) and worldwide (16.4%). Interestingly, although almost 90% of the physicians knew that most RDs are of a genetic character, only half of them correctly assessed the percentage of RDs that are of a genetic basis (50.3%). Similarly, 53.9% of respondents were aware that RDs affect children.

**Table 2:** Physicians’ knowledge about rare diseases

Characteristics	N (%)
Have you ever heard the term ‘rare diseases’?	
Yes	164 (99.4)
No	1 (0.6)
A rare disease is the one that affects less than:	
1 person in 1000	11 (6.7)
** 1 person in 2000**	**30 (18.2)**
1 person in 3000	1 (0.6)
1 person in 5000	13 (7.9)
1 person in 10,000	91 (55.1)
I do not know	19 (11.5)
What is the estimated number of rare diseases?	
100–500	21 (12.7)
1000–2000	34 (20.6)
3000–5000	23 (14)
** 6000**–**8000**	**34 (20.6)**
9000–10,000	4 (2.4)
Over 10,000	28 (17)
I do not know	21 (12.7)
In what age group do rare diseases most frequently appear?	
Newborns	38 (23)
** Children**	**89 (53.9)**
Adolescents	1 (0.6)
Adults	7 (2.4)
They are present in all age groups equally	22 (13.3)
I do not know	8 (4.8)
How many people suffer from rare diseases worldwide?	
10–15,000,000	36 (21.8)
50–75,000,000	23 (13.9)
100–150,000,000	28 (17)
200–250,000,000	10 (6.1)
** 300**–**350,000,000**	**27 (16.4)**
Over 500,000,000	3 (1.8)
I do not know	38 (23)
How many people suffer from rare diseases in Poland?	
500–1000	29 (17.6)
10–15,000	28 (17)
50–75,000	19 (11.5)
100–150,000	20 (12.1)
300–500,000	13 (7.9)
1,000,000	5 (3)
**2**–**3,000,000**	**26 (15.8)**
Over 5,000,000	1 (0.6)
I do not know	24 (14.5)
What is the most common cause of rare diseases?	
Infectious and bacterial	0
** Genetic**	**148 (89.7)**
Autoimmune	8 (4.9)
Mitochondrial	6 (3.6)
Environmental	0
I do not know	3 (1.8)
What percentage of rare diseases are of a genetic origin?	
5–10%	6 (3.6)
20%	18 (10.9)
50%	43 (26.1)
**80%**	**83 (50.3)**
100%	9 (5.5)
I do not know	6 (3.6)

Correct answers are written in bold characters

From the presented list of 28 diseases (including 18 RDs), physicians chose those they considered to be rare (Table 3). Of these, Niemann–Pick disease, Pompe disease and Gaucher disease were the most frequently recognized (79.4%, 76.4% and 73.9% respectively). However, fewer respondents identified properly such commonly known RDs as Duchenne muscular dystrophy (50.3%), neurofibromatosis (37%) fragile X syndrome (52.1%) or phenylketonuria (49.1%). Yet other RDs were recognized by even fewer physicians: cystic fibrosis (40%), achondroplasia (33.9%) or, acromegaly (20%), sickle cell anaemia (8.9%). On the other hand, the most common diseases that were mistaken with RDs turned out to be Munchausen syndrome, fibromyalgia and halitosis.

**Table 3:** Which of the following diseases are considered to be rare in Poland?

	MD N (%)
**Sickle cell anaemia**	13 (8.9)
**Cystic fibrosis**	66 (40)
**Acromegaly**	33 (20)
**Haemophilia**	41 (24.8)
Down syndrome	7 (4.2)
**Niemann–Pick disease**	131 (79.4)
Halitosis	24 (14.5)
Glaucoma	0
**Progeria**	103 (62.4)
**Neurofibromatosis**	61 (37)
**Craniodiaphyseal dysplasia**	78 (47.3)
Cerebral palsy	7 (4.2)
Fibromyalgia	25 (15.1)
**Huntington disease**	94 (57)
**Duchenne muscular dystrophy**	83 (50.3)
Acquired immunodeficiency syndrome	20 (12.1)
Munchausen syndrome	42 (25.4)
**Mucopolysaccharidoses**	104 (63)
**Achondroplasia**	56 (33.9)
Crohn’s disease	10 (6.1)
**Pompe disease**	126 (76.4)
**Gaucher disease**	122 (73.9)
**Fragile X syndrome**	86 (52.1)
**Marfan syndrome**	58 (35.2)
Schizophrenia	1 (0.6)
Alzheimer’s disease	4 (2.4)
**Osteogenesis imperfecta**	97 (58.8)
**Phenylketonuria**	81 (49.1)

Correct answers are written in bold characters

More than three quarters of the respondents agreed that RDs constitute a serious public health issue (83.1%); however, almost 90% of them rated their knowledge about RDs as insufficient or very poor (Table 4). Moreover, 93.4% of physicians did not feel prepared for caring for an RD patient. Simultaneously, over 80% of the respondents were interested in broadening their knowledge of RDs and 76.3% believed that there should be an obligatory course on such diseases in the medical curricula. 69.7% of respondents declared having had classes about RDs during their studies and they declared that the main sources of information about RDs were mandatory courses at the university (46.1%), or scientific literature (38.9%) and the Internet (31.7%). Unsurprisingly, as respondents knew that the majority of RDs are of a genetic character and affect children, most physicians believed that it is mainly geneticists (55%) and paediatricians (53.9%) that should be trained in these diseases. Simultaneously, one fifth of respondents declared that every physician regardless of their specialization, should possess such knowledge and skills (22.4%). However, only 32.1% indicated neurologists. Moreover, even though many RD patients experience mental deterioration, psychiatric and behavioural symptoms, only 10.9% of respondents pointed to psychiatrists as a source of possible help.

**Table 4:** MD’ self-assessment of their knowledge about RDs

	N (%)
Do RDs constitute a serious public health issue?	
Definitely yes	42 (25.5)
Yes	95 (57.6)
No	18 (10.9)
Definitely not	2 (1.2)
I do not know	8 (4.8)
How would you rate your knowledge about rare diseases?	
Very good	1 (0.6)
Fair enough	8 (4.8)
Insufficient	98 (59.4)
Very poor	58 (35.2)
Do you feel prepared for caring for a patient with a rare disease?	
Definitely yes	2 (1.2)
Rather yes	6 (3.6)
Rather not	77 (46.7)
Definitely not	77 (46.7)
I do not know	3 (1.8)
Would you like to broaden your knowledge about rare diseases?	
Yes	137 (83)
No	11 (6.7)
I do not know	17 (10.3)
Do you think that there should be a mandatory course on rare diseases in the medical curricula?	
Definitely yes	38 (23)
Rather yes	88 (53.3)
Rather not	27 (16.4)
Definitely not	1 (0.6)
I do not know	11 (6.7)
Did you/do you have any classes about rare disease during your studies?	
Yes	115 (69.7)
No	39 (21.6)
I do not know	11 (6.7)
Where do you/did you get your knowledge about rare diseases from?	
Mandatory courses at the university	77 (46.1)
Facultative courses at the university	23 (13.8)
Scientific literature and research	65 (38.9)
Scientific conferences, symposia	36 (21.6)
Internet	53 (31.7)
Other	2 (1.2)
I do not search for such information	0
Which physicians should be uniquely trained in RDs?	
Family physician	60 (36.4)
Paediatrician	89 (53.9)
Neurologist	53 (32.1)
Geneticist	92 (55.7)
Psychiatrist	18 (10.9)
Immunologist	45 (27.3)
Other	39 (23.6)
Every physician regardless of their specialization	37 (22.4)

We analysed the responses from Tables 1, 2 and 3 in detail for possible interdependencies. It seems that the studied group of physicians was quite homogeneous, and people of different sex, age and professional experience answered different questions in a similar way. One of the few differences found was in the answer to the question regarding the way of acquiring knowledge of RDs (Table 5). While all physicians declared that the Internet was an important source of information about RDs, respondents over 30 years of age, as well as those with more professional experience, indicated scientific literature, scientific conferences and symposia more often. However, higher statistical significance was obtained in the case of respondents’ age as a variable. A correlation between respondents’ age and years of professional experience of course naturally exists, but in our case it was not linear.

**Table 5:** The odds of using different sources of knowledge on RDs for physicians over and below 30 years

	Medicine doctors over 30 years old versus medicine doctors below 30 years old		
	Odds of using scientific literature and researches to broaden knowledge on RD	Odds of using scientific conferences and symposia to broaden knowledge on RD	Odds of using the Internet to broaden knowledge on RD
OR	2.96	2.79	1.23
95% CI	1.35–6.52	1.01–7.69	0.58–2.60
*p*	**0.004**	**0.02**	0.29

Statistically significant results are written in boldface

*OR* odds ratio, *CI* confidence interval

## Discussion

Delays in the diagnosis of patients with RDs related to deficiencies in doctors’ competence are a serious problem. This is confirmed by reports from patient organizations and researchers dealing with such diseases [3, 39, 40]. Diagnosing many RDs is extremely difficult and sometimes requires the support of the latest biotechnology. It seems, however, that in addition to systemic and organizational circumstances, the key factor responsible for the occurrence of delays in the diagnosis of RDs is the human one. An error or overlooking evidence is often indicated as the cause of misdiagnosis or delays in diagnosis. Unfortunately, recent reports in this area show that not much has changed in this regard in recent years [41–43].

As Cismondi et al. rightly observe, “the academic sector lacks initiatives to improve the knowledge of health professionals on complex topics such as RDs” [44]. The results of this study seem to have largely confirmed the previous findings [45–47] and suggest that healthcare professionals’ in Poland possess insufficient knowledge on RDs and are unprepared to care for RD patients. Similarly, it was shown that also in other countries, i.e. Belgium or Ireland, where GPs tend to be the first caregivers for RD patients, establish a diagnostic referral and have access to information resources on RDs, few physicians possess adequate knowledge on such diseases and rarely use Orphanet or other reliable sources on the Internet [26, 27]. However, this research also shows that physicians are aware of the importance of RDs, as well as of their knowledge deficits and unpreparedness in this regard. It is hard to disagree with Groft et al. [48] that “[a] core foundational method to enhance the study of rare diseases is exposing learners to these conditions during their medical education journey”. Thus, the results of our research confirm that for most physicians university classes were the main source of their knowledge about RDs. However, it was surprising that 21.6% of physicians didn’t receive any classes about RDs during their studies. Even more alarming was respondents self-assessment of their preparedness for caring for RD patients. One should also bear in mind the role of postgraduate training in this area, since research shows that some physicians are interested in this way of training [49].

Another important finding was that most respondents did not believe that all physicians, regardless of one’s medical specialization, should be trained about RDs, as most indicated this function to geneticists and paediatricians. This is significant as a previous study conducted among future physicians showed some important differences [18]. While medical students declared that geneticists (77.4%), paediatricians (75.5%), neurologists (38%) and immunologists (50.3%) should acquire the knowledge and skills necessary to diagnose and care for RD patients more often than the physicians from this study, they also differed significantly in their belief that also family physicians (46.2%) and psychiatrists (22.8%) should be trained with regard to RDs. Thus, while medical students chose almost all the indicated specializations from the list more often than their already professionally active colleagues, the physicians interviewed in this study indicated the option ‘other specializations’ more frequently than the students did. This difference is especially surprising when we consider the significant contribution of family physicians in the diagnosis of RDs [42, 50]. Thus, while we may wonder whether these differences result from physicians’ post-graduation experiences, there is no doubt that such a difference does exist.

A difference of opinions between physicians and medical students [17] was also observed in relation to their interest in broadening their knowledge about RDs and whether there should be a mandatory course on such diseases (Table 6). Physicians not only seemed to be more eager to acquire extra knowledge on RDs but they also saw the need to include the RDs topics into the medical curricula more often. While this is probably due to physicians’ professional experience, it appears that students’ negative opinions on the idea of additional classes on RDs may result from overloading the curricula and not from their assessment of the suitability of an RD course, especially in that both groups agreed that RDs constitute a serious public health issue.

**Table 6:** Odds of being interested in broadening knowledge about RDs and adding a mandatory course on such diseases to the medical curricula

	Medicine doctors versus medical students	
	Odds of not being interested to broaden knowledge on RDs	Odds of being of the opinion that there should be a mandatory course on RDs
OR	0.58	3.84
95% CI	0.36–0.93	2.54–5.82
*p*	**0.01**	**0**

Statistically significant results are written in boldface

*OR* odds ratio, *CI* confidence interval

Considering the fact that RD patients constitute 6–8% of the general population, it is puzzling that only three-quarters of physicians confirm ever meeting a person suffering from an RD. Of course, our respondents worked in various health care units, and this fact may have affected their chance of encountering a person with RD, but not meeting such a patient in several or several dozens of years of work is even theoretically impossible. A recent study at a university hospital in Germany showed that patients with RDs accounts for 19% of all hospitalized patients [51]. It is probably the case that not so many of RD patients appear in other health system facilities, but this does not mean that they are not there at all. We can deal here with the biases in judgment, described by Tversky and Kahneman, which reveal some heuristics of thinking under conditions of uncertainty [52]. In the case of RDs and physicians’ estimations we deal with representativeness and availability heuristics. If the diseases themselves are called rare diseases, and, in addition, for some reason the respondents failed to learn in the course of their medical studies about their frequency, there may be a problem of correctly estimating the chance of encountering a person with such a disease. As Kahneman described later, mistakes in our thinking have their source in ‘the very structure of cognitive mechanisms’ [53]. A recent study showed that in the case of medical students, the mere fact of making them aware during the course of a lecture how often they would meet patients with certain RDs in their professional career causes them to change the assessment of the importance of RDs [54]. Of course, this in no way justifies the delay or misdiagnoses in patients with RDs, it only shows the importance of this knowledge for the process of the proper diagnosis and treatment of rare diseases. There is no doubt that diagnosing RDs can be a significant challenge, but in order to make it possible at all, it is necessary for the physicians to assume that the patients they see may suffer from RDs.

An interesting finding from our study is that the Internet remains an important source of knowledge about rare diseases for doctors. Previous studies have indicated that the Internet is the primary source of information among both students and healthcare professionals [55]; however, in our study doctors with several years’ professional experience declared acquiring such knowledge primarily from specialist literature and scientific conferences. Yet for young physicians the Internet was the primary source of information on RDs, which is probably not surprising, considering that as people only just starting their careers in the medical profession, they do not participate in seminars or conferences as frequently as their older colleagues. However, the observed tendency of many medical journals to reject publications about rare diseases as yielding low citation rates might leave the medical professional insufficiently updated on the progress in this field, should they rely solely on these journals as their source of information on the topic [56].

All in all, while it can be difficult or impossible for a physician to diagnose and select a proper treatment path for a patient with non-specific clinical presentation, for RD patients it is usually confusing, chaotic and expensive and often turns into the long-lasting diagnostic odyssey [3, 28, 31, 33–35, 39, 40, 57]. For example, Libura et al. [3] have shown that while over 61% of RD patients in Poland have heard at least one misdiagnosis before receiving the correct one, on average they face 3.5 misdiagnosis. Moreover, 24.1% received their diagnosis after 10 or more years. Interestingly, from 38.6% of patients who were diagnosed properly during the first consultation, many suffered from RDs that are covered with screening tests: cystic fibrosis and phenylketonuria [3, 58]. This is important because due to lack of GP’s knowledge on RDs before the correct diagnosis is finally made, most patients with rare or ultra-rare diseases consult a number of doctors and often receive many differing opinions, misdiagnoses or late diagnosis. Consequently, they are exposed to many unnecessary treatments, including tests, drugs, hospital readmissions and surgeries. Moreover, many of these procedures are uncomfortable, frightening or painful and may result in the worsening of an RD patient’s condition or even her or his premature death. They also cause additional medical costs for both RD families and the healthcare system.

Thus, although the problem of the diagnostic odyssey is also present in other countries, its seems that also in Poland the prime reason for this is lack of knowledge on RDs among healthcare professionals, including GPs, or lack of access to modern diagnostic methods [59]. Consequently, physicians’ lack of knowledge on RD and its negative impact on the diagnostic process has become the most important argument supporting the changes in medical curricula and public policies for people with RDs. For that reason, one of the strategic points in the recent declaration on RD policy made by the Polish Ministry of Health is the improvement of undergraduate and postgraduate education on RDs by including lectures and seminars on RDs during the last 2 years of studies and by creating a system of postgraduate training offering mandatory specialization training sessions and specialized courses on RDs [60].

At the same time, although lack of knowledge among general practitioners is one of the main reasons why patients suffering from RDs have to face a delay in the diagnosis, it should be also emphasized that Poland struggles with an insufficient number of specialists in the fields of medicine that are crucial from the perspective of RDs, including: clinical genetics, pediatrics, internal medicine, neurology, immunology or child psychiatry. Moreover, although according to the Supreme Audit Office, there are approximately 500 genetic laboratories in Poland, there is still an insufficient number of specialists in the field of medical genetics: in 2017 there were only 122 physicians with specialization in clinical genetics and 194 diagnosticians specialized in the medical genetics laboratory. Consequently, it should not surprise that such a low number of specialists results in a prolonged time for genetic consultation which on average takes 2 years [61].

What is also problematic is that while the number of physicians in Poland in relation to the population is the lowest in the European Union, the country struggles with an inadequate number of primary care physicians and specialists. This, in turn results in a lower efficiency and effectiveness of treatment, i.e. it contributes to choosing subsequent treatments and generates higher costs, especially for RD patients [62]. This is even more important when we consider the fact that although most RDs occur shortly after birth or in early childhood and Poland currently screens for 28 rare diseases [58], out of 70% of young physicians who choose residency, very few opt for specializations that are of particular importance from the perspective of RDs: clinical genetics, pediatrics, internal medicine, neurology, immunology, gynecology or child psychiatry. Thus, even though it is mostly molecular geneticists who perform genetic testing on RDs, physicians that deal with children, the group of patients mostly affected by RDs, should be adequately trained in such diseases.

In addition, it should be stressed that in Poland there is no single RD registry, as there are rather separate registries for different groups of diseases or even a single disease, such as rare neurodegenerative diseases, rare eye diseases, rare skin diseases or rare cancers. However, one of the most important registries where general practitioners can find information on the prevalence of RDs in Poland is the Polish registry of congenital malformations established in 1997 [63]. Moreover, as of 2014, physicians are obliged to report all congenital malformations to this registry. Its central management and database is located at the Department of Medical Genetics at PUMS, Poland.

## Limitations

Although this study brings a new insight into the how much Polish physicians are acquainted with RDs, its findings are limited in several respects that may impact their generalizability and interpretation. First, although the response rate was reasonably high, medical doctors taking their specialization courses from only one Polish medical university were enrolled in the study. Second, as some physicians refused to complete the questionnaire, the results represent solely the opinions of those who agreed to participate in the study and cannot be generalized for the entire population of general practitioners either in Poznan or in Poland as a whole. Consequently, more in-depth studies would be required. Third, although it would be important to know the features of those GPs who refused to take part in the study, since it could allow better profiling of future educational actions, as to the anonymity of our survey, it was impossible to identify those physicians who rejected the invitation. However, some advantages of this study should also be acknowledged. Most importantly, as there is a scarcity of previous work on the knowledge of Polish physicians about RDs this study may stimulate further research on the physicians’ educational needs related to diagnosis, management and treatment of RDs.

## Conclusions

All in all, while this study has identified the gap in physicians’ knowledge on RDs, we believe that their empowerment is a huge project that requires implementation of multilevel solutions. Thus, we suggest that in order to overcome the deficits in physicians’ knowledge on RDs and address their educational needs the following guidelines should be implemented:

1. Because medical education programs are a great way of raising awareness and interest in RDs, university curricula in Poland should include mandatory teaching programs on RDs. However, because it is impossible to teach future physicians about each and every RD, it seems that in the first place students should be familiarized with the more common RD as it would allow them to recognize them more rapidly in clinical practice. In addition, students should be taught how to differentiate RD from more common conditions and what actions should be taken when an RD is suspected.
2. Apart from the existing training in molecular biology, diagnostic laboratory and clinical genetics, future physicians should be also trained in genetic counselling which would help them to understand not only the nature of genetic disorders but also what the test results will mean for the patient and one’s family, what the limitations of genetic testing are and what treatment and support options are available for RD patients. Medical students should be also trained in clinical genetics ambulatories.
3. To raise students’ awareness on the problems that patients suffering from RDs have to deal with, patient organizations and advocacy groups could be involved. Such direct contact with RD patients and their caregivers would help future physicians understand better the challenges persons with RDs face on a daily basis, including the diagnostic pathway, access to the referral system or orphan drugs.
4. During their examinations, including the National Medical Exam, future physicians should be asked some questions referring to RDs. However, due to the reasons mentioned above, such questions should focus on students’ diagnostic reasoning, procedure, and management.
5. Postgraduate specialization courses on RDs for physicians and other healthcare workers should be also organized. However, because most physicians lack time and the Internet is the main source of information on RDs, e-learning modules should be organized. They should include tutorial lecturer videos, webinars and PowerPoint presentations.
6. It would be advisable to create a digital application or a specialized information platform with reliable information on standardized and detailed guidance on diagnosis, clinical standard guidelines, referral, counselling, drugs reimbursement, treatment options and centers and the medical insurance system specified for RD.
7. Finally, because most RDs require multidisciplinary approach, an integrated medical service platform should be established to facilitate better communication between various specializations.

## Data Availability

The datasets generated during and/or analyzed during the current study are available from the corresponding author on reasonable request.

## Abbreviations

RD: Rare disease
EU: The European Union
CEE: Central Eastern Europe
PUMS: Poznan University of Medical Sciences
GPs: General practitioners
